# Copy Number Variations and Schizophrenia

**DOI:** 10.1007/s12035-022-03185-8

**Published:** 2022-12-29

**Authors:** Kamila Szecówka, Błażej Misiak, Izabela Łaczmańska, Dorota Frydecka, Ahmed A. Moustafa

**Affiliations:** 1 Department of Genetics, Wroclaw Medical University, Marcinkowskiego 1, 50-368 Wroclaw, Poland; 2 Department of Psychiatry, Wroclaw Medical University, Pasteura 10, 50-367 Wroclaw, Poland; 3 Department of Human Anatomy and Physiology, The Faculty of Health Sciences, University of Johannesburg, Johannesburg, South Africa; 4 School of Psychology, Faculty of Society and Design, Centre of Data Analytics, Bond University, Gold Coast, Queensland Australia

**Keywords:** Schizophrenia, Genetics, Copy number variations (CNVs), Neural studies

## Abstract

Schizophrenia is a neurodevelopmental disorder with genetic and environmental factors involved in its aetiology. Genetic liability contributing to the development of schizophrenia is a subject of extensive research activity, as reliable data regarding its aetiology would enable the improvement of its therapy and the development of new methods of treatment. A multitude of studies in this field focus on genetic variants, such as copy number variations (CNVs) or single-nucleotide variants (SNVs). Certain genetic disorders caused by CNVs including 22q11.2 microdeletion syndrome, Burnside-Butler syndrome (15q11.2 BP1-BP2 microdeletion) or 1q21.1 microduplication/microdeletion syndrome are associated with a higher risk of developing schizophrenia. In this article, we provide a unifying framework linking these CNVs and their associated genetic disorders with schizophrenia and its various neural and behavioural abnormalities.

## Introduction

Schizophrenia is a chronic and complex mental disorder that affects about 0.5% of the population [1]. Patients usually present first symptoms of schizophrenia around the age of 16–30 years [2, 3]. Schizophrenia is mainly characterized by relapsing episodes of psychosis; however, occurring symptoms can be divided into two main categories: positive and negative symptoms. Positive symptoms include various types of hallucinations and delusions. In turn, negative symptoms include social withdrawal, diminished emotional range, avolition, anhedonia and alogia. There is accumulating evidence that specific dimensions of psychopathology in schizophrenia might be characterized by distinct neurobiological mechanisms. For instance, Carpenter et al. [4] differentiated the deficit subtype of schizophrenia, which is characterized by primary and enduring negative symptoms, from non-deficit schizophrenia, which is characterized by mild negative symptoms. Further studies have demonstrated that patients showing deficit and non-deficit schizophrenia differ in clinical characteristics, neurobiological features, risk factors and family history [5–8]. Unlike non-deficit schizophrenia, deficit schizophrenia is associated with alterations of the brain structure, brain activation, worse sensory integration and impaired motor coordination. The existence of a significant relationship between deficit schizophrenia and more severe cognitive impairment has also been suggested [9–14].

Numerous mental disorders, including schizophrenia, have multifactorial backgrounds based on complex interactions between genetic and environmental factors [5]. Among known genetic alterations reported in patients with schizophrenia, copy number variants (CNVs) are one of the most often genetic alterations that might be causative. There are some reports on the presence of the highest burden of larger (> 500 kb) exonic CNVs in schizophrenia and the possibility that carrying these alterations may modify the phenotype in patients at risk of schizophrenia [15–18]. The phenotype of patients carrying CNVs might include schizophrenia as the only clinical manifestation. However, in other cases, schizophrenia develops as a part of known genetic syndromes attributable to CNVs, such as 1q21.1 microdeletion/microduplication syndrome, 15q11.2 BP1-BP2 microdeletion syndrome or 22q11.2 deletion syndrome [19–22]. The development of the array comparative genomic hybridization (aCGH) and the next-generation sequencing (NGS) methods together with an array of bioinformatic approaches has largely improved our understanding of the association between CNVs and various neurodevelopmental disorders, including schizophrenia [23].

CNVs can be defined as alterations of the number of copies in specific regions of the genome (chromosomal duplications and deletions), which vary between individuals. Notably, CNVs represent 4.8–9.5% of the human genome and have been observed to exert an effect on the expression, as well as the function of genes [24]. It has been demonstrated that approximately 70% of all individuals carry at least one rare CNV. Among them, deletions are less common than duplications [25–28].

Existing methods for detecting CNVs in the genome are typically based on DNA probes, such as the multiplex ligation-dependent probe amplification (MLPA), aCGH or NGS. Results for alterations above 40 kbp can also be verified using the fluorescence in situ hybridization (FISH). In recent years, there has been a rapid technological development of molecular biology methods, which is why an increasing use of aCGH and NGS is observed both in the diagnostics of SNVs and CNVs. However, in many countries, this is still limited by the relatively high cost of the test and the high skill requirements of personnel [29].

## CNVs in Schizophrenia

**Fig. 1:**
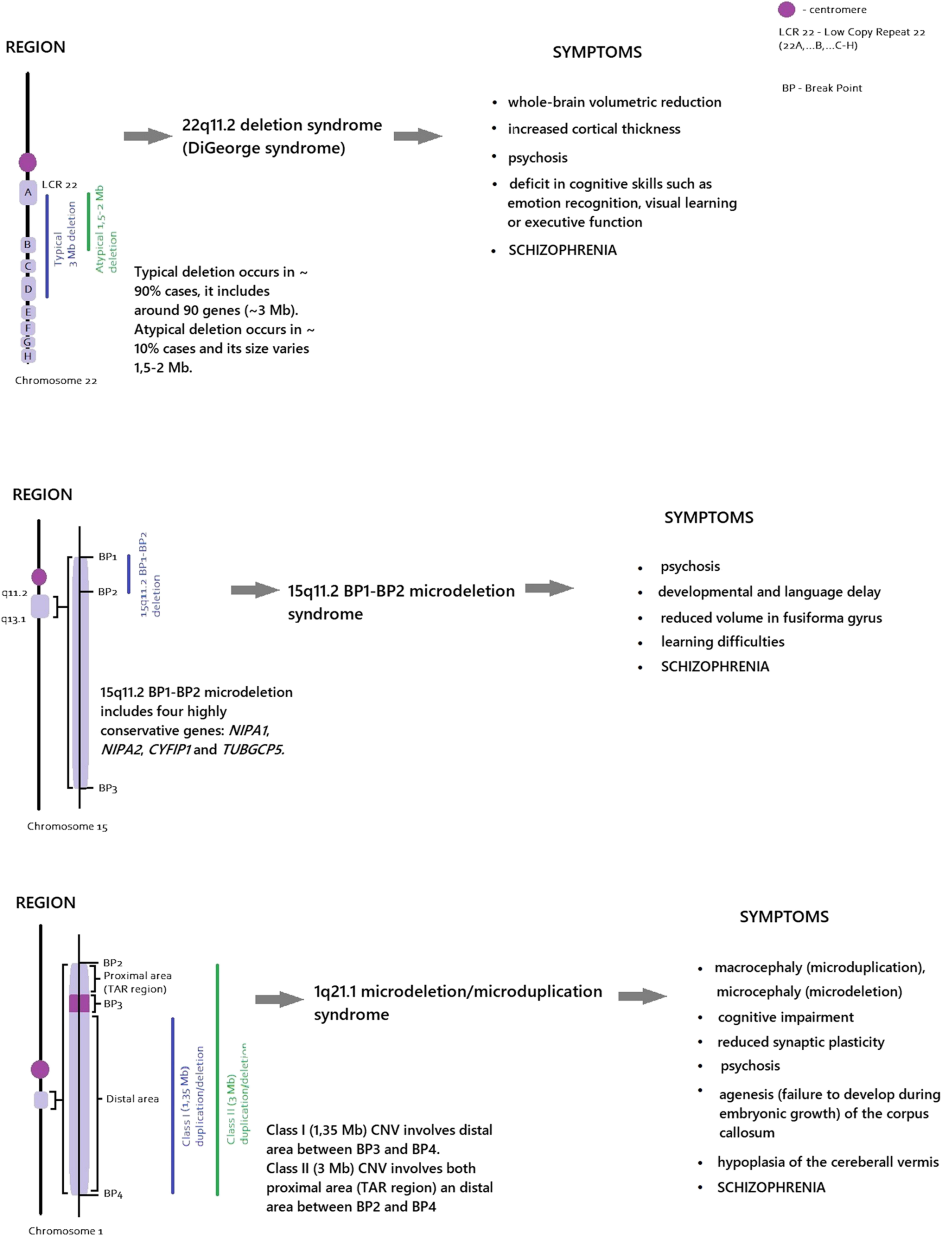
Simplified overview of the phenotype and specific genes associated with CNVs located at 22q11.2, 15q11.2 and 1q21.1. p. 12

However, CNVs associated with schizophrenia lack diagnostic specificity, that is, the presence of certain CNVs in the genome can increase a risk of several other neurodevelopmental disorders, including autism spectrum disorder, attention-deficit hyperactivity disorder and intellectual disability [30–32]. According to recent studies, schizophrenia has been associated with several recurrent CNVs, which are major risk factors of the disorder, occurring in 3.5–5% of cases (see Table 1 and Fig. 1) [20, 33, 34]. However, due to the fact that those CNVs are not completely penetrant (reduced penetrance) and expressed on different levels (variable expressivity), they are not sufficient to cause schizophrenia by themselves. The percentage of individuals with CNVs who develop symptoms may vary depending on the type of CNVs and other factors (i.e., those related to interactions with other genes and the impact of environment) [35].

**Table 1:** Overview of selected genetic syndromes associated with schizophrenia. p. 6, 7

Disorder	Region/genes	Symptoms	Breakpoint location
1q21.1 microdeletion/microduplication syndrome	Class I (1.35 Mb) CNV involves distal area between BP3 and BP4 Class II (3 Mb) CNV involves both proximal area (TAR region) and distal area between BP2 and BP4	- Macrocephaly (microduplication), microcephaly (microdeletion) [38] - Cognitive impairment [38] - Reduced synaptic plasticity [38] - Psychosis [38] - Agenesis (failure to develop during embryonic growth) of the corpus callosum [39] - Hypoplasia of the cereberall vermis [39]	Class I deletion: 1q21.1 BP3-BP4 Class II deletion: 1q21.1 BP2-BP4
15q11.2 BP1-BP2 microdeletion syndrome	This type of 15q11.2 microdeletion includes four highly conservative genes: *NIPA1*, *NIPA2*, *CYFIP1* and *TUBGCP5*	- Psychosis [40] - Developmental and language delay [40] - Reduced volume in fusiforma gyrus [41] - Learning difficulties[42]	15q11-q13
22q11.2 deletion syndrome	Typical deletion occurs in ~ 90% cases, and it includes around 90 genes (~ 3 Mb) Atypical deletion occurs in ~ 10% cases, and its size varies 1.5–2 Mb	- Whole-brain volume reduction [43, 44] - Increased cortical thickness [45] - Psychosis [46] - Deficit in cognitive skills such as emotion recognition, visual learning or executive function [47, 48]	Typical deletion: 22q11.2 (LCR22A–LCR22D) Atypical deletion: 22q11.2 (LCR22A-LCR22B)

Interestingly, CNVs associated with schizophrenia have also been observed in healthy individuals, and thus, it may suggest that either environmental factors or additional genetic abnormalities contribute to the development of schizophrenia. Moreover, some genetic syndromes such as 22q11.2 microdeletion syndrome, Burnside-Butler syndrome (15q11.2 microdeletion) or 1q21.1 microduplication/microdeletion are strongly associated with a risk of schizophrenia among other CNVs [34, 36, 37]. Table 1 and Fig. 1 show an overview of clinical characteristics of these genetic syndromes.

In the next subsections, we review genetic syndromes commonly associated with a risk of schizophrenia. Recent studies report that other CNVs can also be associated with a higher risk of schizophrenia. Those CNVs (listed in Table 2) include deletions and duplications on chromosomes 2, 3, 7, 8, 9, 13, 16 and sex chromosome X [20, 49].

**Table 2:** The comparison of schizophrenia-associated CNV *loci* according to “Schizophrenia-associated genomic copy number variants and subcortical brain volumes in the UK Biobank” [20] and “Contribution of copy number variants to schizophrenia from a genome-wide study of 41,321 subjects” [65]. Only two publications were used as sources to create the said table, as they contained the most information about multiple CNVs compared to others. p. 14, 15

Schizophrenia-associated CNV *loci* according to Warland et al., 2019	Schizophrenia-associated CNV *loci *according to Marshall et al., 2017
Deletions	Deletions
1q21.1	1q21.1
2p16.3 (*NRXN1*)	2p16.3
3q29	3q29
-	7q11.21 (*ZNF92*)
-	7p36.3 (*VIPR2*; *WDR60*)
-	8q22.2 (*VPS13B*)
-	9p24.3 (*DMRT1*)
15q11.2	15q11.2
15q13.3	15q13.3
16p12.1	-
-	16p11.2 (*distal*)
22q11.2	22q11.2(1)
Duplications	Duplications
1q21.1	1q21.1
-	7q11.21 (*ZNF92*)
7q11.23	7q11.23
-	7p36.3 (*VIPR2*; *WDR60*)
-	9p24.3 (*DMRT1*)
-	13q12.11 (*ZMYM5*)
15q11-q13	-
16p11.2	16p11.2 (*proximal*)
16p13.11	-
-	22q11.21
-	Xq28 (*distal*)
-	Xq28 (*MAGEA11*)

### 15q11.2 Microdeletion Syndrome

Burnside-Butler syndrome is a neurodevelopmental disorder with a genetic basis, i.e., the occurrence of this syndrome is correlated with the presence of pathogenic CNV. Symptoms of Burnside-Butler syndrome include altered brain morphology, cognitive impairment and behavioural alterations. This disease is caused by the 15q11.2 BP1-BP2 deletion, which is a rare CNV spanning about 500 kbp. This region includes four highly conservative genes, including *TUBGCP5*, *CYFIP1*, *NIPA1* and *NIPA2* that are expressed in the brain. The dysfunction of their protein products is also associated with Prader-Willi syndrome (PWS) and Angelman syndrome (AS). The 15q11.2 BP1-BP2 deletion is present in 0.57–1.27% of the world population [50]. Inter-individual variability in clinical manifestation might be the consequence of incomplete penetrance and variable expression levels of genes located in the 15q11.2 BP1-BP2 region [40, 50].

Apart from CNVs covering the above-mentioned genes, the presence of pathogenic variants, commonly referred to as single nucleotide variants (SNVs), is reported as the mechanism underlying the development of Burnside-Butler syndrome which has been previously reported [40, 50].

### 22q11.2 Microdeletion Syndrome

Around 30 years ago, it was demonstrated that schizophrenia tends to co-occur with velocardiofacial syndrome [51]. In the same year, both velocardiofacial syndrome and 22q11.2 microdeletion syndrome (formerly known as DiGeorge syndrome), two clinical syndromes that were later classified into the spectrum of one syndrome of congenital abnormalities, were associated with deletions of the 22q11.2 region [52, 53]. However, the first genome-wide analysis of CNVs in patients with schizophrenia was published in 2007 [54].

The 22q.11.2 deletion occurs approximately in 1 of every 4000 live births and is equally distributed between males and females. The 22q11.2 microdeletion syndrome is associated with clinical manifestations with considerable interindividual variability; however, most frequently, they include immunodeficiency, congenital cardiac anomalies and palatal abnormalities (each one being present in ~ 75% of individuals diagnosed with 22q11.2 microdeletion syndrome) [55]. The prevalence of schizophrenia in subjects with 22q11.2 microdeletion syndrome has been estimated at 25%, and this syndrome is known to be the strongest genetic factor of schizophrenia [56].

Major microdeletions, causative for the syndrome, are classified as CNVs and are usually larger than 1 kb. The inheritance of 22q11.2 microdeletion syndrome is autosomal dominant, as deletion of one copy chromosome 22 is sufficient to cause the disorder. However, most frequently, the 22q11.2 microdeletion occurs spontaneously during gametogenesis. Parental studies show no association of the deletion with age of the parents [57–60]. This hemizygous deletion includes typically around 90 genes (~ 3 Mb). Parental studies show that the deletion inheritance occurs approximately in 10% of cases, while ~ 90% are caused by random deletion during gametogenesis or early development of the foetus [61]. Most of 22q11.2 microdeletions (~ 90%) are approximately 3 Mb in size; the remaining 10% include a nested deletion (~ 1.5–2 Mb) or atypical, non-nested distal microdeletions. Recent studies on CNVs in schizophrenia demonstrated that 5% of cases showed associations with rare CNVs rather than common CNVs. Furthermore, individuals with previously mentioned rare CNVs are about twenty times more likely to develop schizophrenia, indicating that the 22q11.2 microdeletion should be considered when diagnosing schizophrenia [26, 62, 63] (Table 1). Notably, microdeletions of various sizes in the 22q11.2 region also predispose to the development of other neurodevelopmental disorders, including attention-deficit/hyperactivity disorder (ADHD) and autism spectrum disorder [57, 64].

### 1q21.1 Microdeletion/Microduplication Syndrome

The 1q21.1 region contains many low-copy repeat sequences, and thus, it is susceptible to deletions and duplications occurring during meiotic division. The 1q21.1 locus is divided into four segment breakpoints (BP): BP1, BP2, BP3 and BP4. Therefore, two classes of the 1q21.1 microdeletion/microduplication-related CNVs have been described and include smaller 1.35 Mb class I CNVs and larger ~ 3 Mb class II CNVs. Class I CNVs involve the segment between BP3 and BP4, which is localised in the distal part of the 1q21.1 region. Class II microdeletion/microduplications are placed from the distal 1q21.1 to the proximal 1q21.1 regions, between BP2 and BP4 [17, 65]. Both microdeletions and microduplications can occur de novo or can be inherited in an autosomal dominant manner. Importantly, CNVs in the 1q21.1 region are characterized by incomplete penetration and variable expression [66].

The proximal part of the 1q21.1 region includes the *RBM8A* gene that is causally associated with the thrombocytopenia-absent radius (TAR) syndrome. However, microdeletions covering the distal part of the 1q21.1 region may manifest in developmental disorders, microcephaly and schizophrenia [67]. Many patients with the 1q21.1 microduplication show other signs of psychopathology, including anxiety, depression and ADHD. Neurological manifestations that include epilepsy and hypotonia might also occur leading to developmental delay. In paediatric patients, microduplication in this region might be associated with macrocephaly, developmental delay and autism spectrum disorder. In turn, the 1q21.1 microdeletions are predominant in patients diagnosed with schizophrenia and microcephaly [67].

## Association Between CNVs and Clinical Features of Schizophrenia

There are many possibilities of how the different CNVs contribute to the development of schizophrenia and its numerous clinical features, including deficits in social skills, learning processes, emotional recognition and cognitive flexibility [47, 63, 68–70]. Moreover, it is reported that patients with schizophrenia and pathogenic CNVs are more likely to present treatment resistance [71]. The mechanism of how CNVs influence symptoms of schizophrenia remains unclear. However, mouse models and induced pluripotent stem cells have successfully been used by studies addressing the neurobiology of CNVs associated with schizophrenia [72–77]. Abnormalities of basic associative learning processes have long been correlated with this particular disorder. These observations have been followed by many researchers, including studies conducted in 2017, results of which show that CNVs impact inhibitory learning in schizophrenia, which potentially contributes to the development of core symptoms in this disorder [63]. Genes associated with processes involved in synaptic plasticity (such as genes encoding components of the NMDA receptor complex, connected with glutamatergic signalling, but also genes involved in inhibitory GABAergic modulation of neuronal signalling) have been shown to be affected by CNVs [78, 79]. Products of these genes are synaptic proteins regulating the molecular processes directly related to associative learning and memory. Deficits in associative learning in individuals with schizophrenia have been assumed to contribute to the development and persistence of psychotic and cognitive symptoms. Patients with schizophrenia typically show the persistence of delusional beliefs, and thus, impairment of extinction learning could be an explanation of the core symptoms of schizophrenia [19, 33, 80, 81].

The study conducted in young patients diagnosed with 22q11.2 deletion syndrome has demonstrated that both negative and positive symptoms of schizophrenia were escalated in individuals with worst social skills. In the study from 2016, symptoms typical for schizophrenia, such as social anxiety and lack of close friends, were found to be increased in individuals with 22q11.2 DS [68–70].

22q11.2 deletion and other high-risk CNVs also contribute to a large extent to the intellectual disability, which was proved in a study using genome-wide microarrays. Moreover, scores of attention tests correlate with particular symptoms of schizophrenia. Lower scores are associated with negative symptoms, but not positive symptoms in this disorder. Also, low IQ test scores achieved by children can predict the likelihood of developing schizophrenia during adulthood. Lower scores for social and cognitive tests are frequent in young individuals at risk mental states, compared with controls. All mentioned low scores provide probabilistic, not categorical, predictors for schizophrenia development [34, 61, 62, 82, 83].

## Single Nucleotide Variants (SNVs) and Gene Expression in Schizophrenia

Studies on single nucleotide variants in schizophrenia are crucial for understanding the pathological mechanism of this condition. This subject has been discussed previously in details by several authors [22, 84–91]. Two different mechanisms (deletion and point mutations) for loss of function observed for genes critical to any condition with genetic background confirm their clinical significance and support the hypothesis about double-hits while loss of heterozygosity (LOH) co-occurring with a functional variants may be relevant [85].

## Neurostructural Abnormalities Associated with Schizophrenia Risk CNVs

Schizophrenia risk CNVs have been noticed to have correlation with certain morphological brain abnormalities. Especially early-onset schizophrenia patients have been noticed to show dysmorphic features. Early detection of individuals with high risk of schizophrenia development is crucial for creating suitable prevention and/or therapy [92]*.*

Windows of vulnerability occurring multiple times during the brain development are when abnormalities might progress which can lead to the development of the symptoms of mental disorders. One of the most popular neurodevelopmental theories in schizophrenia pathogenesis is the two-hit model, which states that there are two points of aberrant development (early brain development and adolescence) that significantly increase the risk for schizophrenia-like symptoms in the individual [93].

The study conducted by Warland et al. (2019) focused on the association of schizophrenia CNVs with subcortical brain volumes [20]. The authors used samples of participants from the UK Biobank. The analysis of MRI data focused on 15 metrics of subcortical volumes, out of which 5 brain regions (right thalamus, left putamen, right pallidum, right hippocampus and right accumbens) presented the significant association with schizophrenia-related CNVs. All of these subcortical structures showed a reduction in volume in patients with schizophrenia and CNVs [20]. Reduced volume of subcortical structures in patients carrying schizophrenia-related CNVs compared to unaffected CNVs carriers were also reported by previous studies [94, 95]. Reduction in volumes of subcortical structures, such as the hippocampus and thalamus, was observed in individuals with high risk of developing schizophrenia, compared to healthy controls [96]. Changes in brain cortical anatomy associated with the presence of rare CNVs have been observed by Caseras et al. in 2021 [97]. The 1q21.1 deletion and the 15q11.2 deletion CNVs were associated with reduced gyrus surface area in carriers. Also, it appeared that the 15q11.2 deletion correlates with thicker cortex in carriers [97]. Finally, the 22q11.2 microdeletion has been associated with thicker cortex and reductions in the cortical surface area [98].

## Discussion

The correlation of selected CNVs at specific *loci* with risk factors for several neuropsychiatric disorders, such as schizophrenia, autism spectrum disorder, intellectual disability and depression, has been reported previously [21, 31, 99]. Further research is needed to determine which genetic abnormalities or genetic variants are related to the phenotypic expression of schizophrenia symptoms, including negative, positive and cognitive abnormalities. For example, the *BCL9* gene polymorphisms are thought to be associated with negative symptoms in schizophrenia, as mentioned gene product is involved in the Wnt signalling pathway, a conserved pathway regulating crucial processes of cell fate determination in metazoan animals, including humans, which is significant in neuroplasticity, neurogenesis and cell survival [46, 100–103].

Genetic background for schizophrenia should not be considered without other crucial aspects of the expressed phenotype—such as differences in synaptic signalling and plasticity, abnormalities in micro and macrostructures of the brain or even environmental factors, which are significant in many psychiatric disorders. Determining which genes are involved in the development of schizophrenia is the first step of determining the role of mentioned genes’ products in molecular pathways leading to the expression of schizophrenia symptoms. The phenotype may also depend on the type of particular CNVs—microdeletion and microduplication phenotypes often appear to be on the two ends of the spectrum. This situation is especially apparent in patients with 1q21.1 microdeletion/microduplication syndrome. Microdeletion in this region is correlated with schizophrenia and microcephaly; microduplication, on the other hand, is prevalent in individuals with autism spectrum disorder and macrocephaly [38].

There are also some reports on 3q29 deletion syndrome which is connected with > 40-fold higher risk for schizophrenia and the presence of treatment-resistant psychotic symptoms, multiple medical comorbidities and early-onset dementia [104, 105], 16p11.2 duplication and [106] and 17q12 deletion [107].

In some cases, like in Burnside-Butler syndrome, the clinical phenotype of the child depends on the origin of parental deletion—if deletion is inherited from the father, there is a higher risk of congenital heart defects in the offspring; however, if deletion is maternal then the risk for intellectual disability and autism is increased [108]. In this situation, awareness of the genetic profiles of the parents can determine abnormalities which their future offspring is more prone to, and it can also increase the quality of genetic counselling.

## Conclusions and Futures Directions

Numerous disorders with a genetic background such as previously mentioned 22q11.2 microdeletion syndrome, Burnside-Butler Syndrome (15q11.2 BP1-BP2 microdeletion) or 1q21.1 microduplication/microdeletion syndrome have been noticed to contribute to a higher risk of schizophrenia development. Patients with said disorders often display psychiatric (i.e., cognitive impairment, psychosis) and neurostructural symptoms (i.e., brain volumetric reduction, increased cortical thickness) that are observed in schizophrenic patients which further highlights the importance of CNVs in the understanding of the genetic background of schizophrenia [38, 40, 43, 109].

It is worth acknowledging that many CNVs occur among the human population and show no pathogenic impact, while others can be associated with a predisposition to various psychiatric disorders. Only 3.5–5% of individuals with schizophrenia are carriers of major-risk CNVs. Moreover, mentioned CNVs have also been observed in healthy individuals, which may suggest that other factors such as epigenetics or environmental conditions could be involved in schizophrenia development [34, 36].

Further research of CNVs that are associated with the development of schizophrenia could contribute to new prospects of therapies and prevention in people with a higher genetic risk of the disorder. More GWAS analyses could also reveal novel copy number variations associated with schizophrenia which, combined with current knowledge about single-gene copy number variants and gene expression, could be vital in the understanding of the aetiology of this psychiatric disorder.

The application of genome–wide experiments not only allows us to better understand the genetic background of schizophrenia but also supports the thesis about its polygenic inheritance and genetic overlap with other mental disorders including autism spectrum disorder or bipolar disorder. A thorough understanding of the genetic basis of this disorder could enable predictive testing, early diagnosis and more effective therapy. Due to the complexity of schizophrenia and a variety of underlying genetic mechanisms, including CNVs, this appears to be a difficult task, but a promising next step may be to apply machine learning techniques together with high-throughput gene research technologies and clinical data analysis.

It is also important to note the complexity of schizophrenia in terms of exposure to environmental factors known to affect a risk of psychosis, clinical manifestation, course of the disorder and clinical and functional outcomes. Moreover, certain aspects of psychopathological symptoms and behavioural abnormalities, which are known to be present in schizophrenia, cross traditional diagnostic boundaries established by ICD and DSM classification systems [110]. These include positive, negative, mood and disorganization symptoms and cognitive impairment [8, 111]. In this regard, future studies that aim to provide new insights into the role of CNVs need to deconstruct the psychosis spectrum by dissecting specific dimensions of psychopathology or symptom clusters. Studies in this field may also use existing approaches related to the Research Domain Criteria (RDoC) and the Hierarchical Taxonomy of Psychopathology (HiTOP) [111, 112]. Also, investigating CNVs with respect to specific endophenotypes that capture phenotype constructs characterized by the association with illness in the population, heritability, state-independent manifestation (expression of the phenotype independent of the illness activity) and the association with illness in families might be helpful in addressing the heterogeneity of the diagnostic construct called schizophrenia [113, 114]. Nevertheless, large samples of individuals with psychosis spectrum disorders will be needed due to relatively low effect size estimates of specific CNVs. However, these research efforts might uncover on how CNVs build up specific phenotypes at the continuum of psychosis.

## Data Availability

Data sharing is not applicable to this article.
